# Are Physiologically Based Pharmacokinetic Models Reporting the Right C_max_? Central Venous *Versus* Peripheral Sampling Site

**DOI:** 10.1208/s12248-015-9796-7

**Published:** 2015-06-23

**Authors:** Helen Musther, Katherine L. Gill, Manoranjenni Chetty, Amin Rostami-Hodjegan, Malcolm Rowland, Masoud Jamei

**Affiliations:** Simcyp Limited (a Certara Company), Blades Enterprise Centre, John Street, Sheffield, S2 4SU UK; Centre for Applied Pharmacokinetic Research, Manchester School of Pharmacy, University of Manchester, Manchester, UK

**Keywords:** C_max_, intravenous, PBPK modelling, pharmacokinetics, physiological model

## Abstract

Physiologically based pharmacokinetic (PBPK) models can over-predict maximum plasma concentrations (C_max_) following intravenous administration. A proposed explanation is that invariably PBPK models report the concentration in the central venous compartment, rather than the site where the samples are drawn. The purpose of this study was to identify and validate potential corrective models based on anatomy and physiology governing the blood supply at the site of sampling and incorporate them into a PBPK platform. Four models were developed and scrutinised for their corrective potential. All assumed the peripheral sampling site concentration could be described by contributions from surrounding tissues and utilised tissue-specific concentration-time profiles reported from the full-PBPK model within the Simcyp Simulator. Predicted concentrations for the peripheral site were compared to the observed C_max_. The models results were compared to clinical data for 15 studies over seven compounds (alprazolam, imipramine, metoprolol, midazolam, omeprazole, rosiglitazone and theophylline). The final model utilised tissue concentrations from adipose, skin, muscle and a contribution from artery. Predicted C_max_ values considering the central venous compartment can over-predict the observed values up to 10-fold whereas the new sampling site predictions were within 2-fold of observed values. The model was particularly relevant for studies where traditional PBPK models over-predict early time point concentrations. A successful corrective model for C_max_ prediction has been developed, subject to further validation. These models can be enrolled as built-up modules into PBPK platforms and potentially account for factors that may affect the initial mixing of the blood at the site of sampling.

**Electronic supplementary material:**

The online version of this article (doi:10.1208/s12248-015-9796-7) contains supplementary material, which is available to authorized users.

## INTRODUCTION

It has been observed that physiologically based pharmacokinetic (PBPK) models can often report over-predicted maximum plasma concentration (C_max_) values compared to *in vivo* data. This is most obvious when administration is *via* the intravenous (i.v.) route over a short time period, for example, a fast infusion or bolus. It is suggested that this discrepancy, apart from practical limitations in drawing blood samples immediately after giving an i.v. dose, may be because PBPK models traditionally report the concentration in the central venous compartment, *i.e.* the “pooled venous return”, whereas in clinical studies, sampling is usually taken from a peripheral vein in the arm due to ethical and logistical considerations (1). These two sites may not have the same concentrations, particularly at early time points where distribution throughout the body tissues is ongoing, and equilibration has not occurred.

With PBPK models increasingly used in assessments of pharmacokinetics, pharmacodynamics and toxicology, this is an issue that cannot be dismissed. PBPK approaches have been used in toxicology for the development of exposure limits (1) and to obtain predicted C_max_ values to be utilised in the assessment of potential adverse effects (2). These models are also increasingly used in pharmaceutical regulatory submissions (3–6). For modelling of pharmacodynamics, if arterial concentrations are considered to drive an observed effect, it is important to understand how this concentration relates to that observed from the clinical venous sampling. It is clearly critical to know what is being reported from PBPK models and how the underlying models relate to the *in vivo* situation.

The idea that over-prediction of C_max_, when reporting from the central venous compartment, is indicative of physiological differences between the central venous compartment and the peripheral sampling site is not new and not only theoretical; evidence to support the concept of local differences in distribution, mixing and concentration between potential sampling sites *in vivo* has been published. Concentration differences can be observed between arterial and venous sampling and can also be dependent on the location of the specific vessels within the body as reviewed by Chiou (7, 8) as early as 1989. This review highlighted numerous examples where the choice of sampling site can lead to significant differences in the observed concentrations, both in human and in animal studies. The author also highlighted that utilising the “wrong” observed concentration could have potentially serious implications.

Differences between the arterial and venous concentrations and potential models for inter-conversion of reported data have been described by a number of investigators (9–14). Many of these publications focus on the implications of these concentration differences for modelling of pharmacodynamics, and these models are generally empirical or compartmental in structure and are not easily applied to PBPK models. In addition, some published models require prior knowledge of *in vivo* concentration-time or pharmacokinetic data, and/or parameter fitting, meaning that they could not be applied in a predictive scenario such as the estimation of first-in-man pharmacokinetics. A physiologically based recirculatory model for fentanyl was constructed and presented by Upton *et al.* (15). However, a number of the parameters were determined using prior knowledge of the compound distribution and hence are not applicable in a generic “bottom-up” PBPK model. Levitt (16) provides the most pragmatic method to address the problem, by using an approach of creating a PBPK model for the arm to describe a peripheral sampling site. This model was developed to describe and predict the kinetics of organic solvents and may not be relevant for a wider variety of pharmaceutical compounds; although it does provide a useful starting point for model development.

The objective of this study was to develop and evaluate a potential corrective model to describe the C_max_ at a peripheral sampling site, utilising the full PBPK model within the Simcyp population-based Simulator. A number of different options were assessed and compared to observed *in vivo* data. The model giving the best performance was selected for further validation and future development.

## MATERIALS AND METHODS

### Model Definitions

The base model in this study follows the assumption that the tissues surrounding a peripheral sampling site contribute, to varying degrees, to the observed venous concentration at that location. A similar approach was suggested by Levitt (16), where “flow fractions” were used to describe the contribution of each tissue to the final overall concentration at the sampling site. These flow fractions were determined using a fitting approach where the values were adjusted within the PBPK model to recover the observed experimental data. The model described herein utilises the tissue concentration profiles predicted by the Simcyp Simulator full PBPK model (17), which allows simulation of tissue concentration data for all organs incorporated within the model. These tissue profiles, which are commonly reported with respect to blood, in combination with the tissue:plasma partition coefficient (Kps), can be used to calculate the emergent concentrations which leave the tissues. These can then be used in combination with drug data and the defined fractions to describe an overall concentration-time profile at a peripheral sampling site where a corrected C_max_ value can be observed. The fractions determined by Levitt (16) provided the initial estimates that were incorporated in the model for validation.

**Fig. 1:**
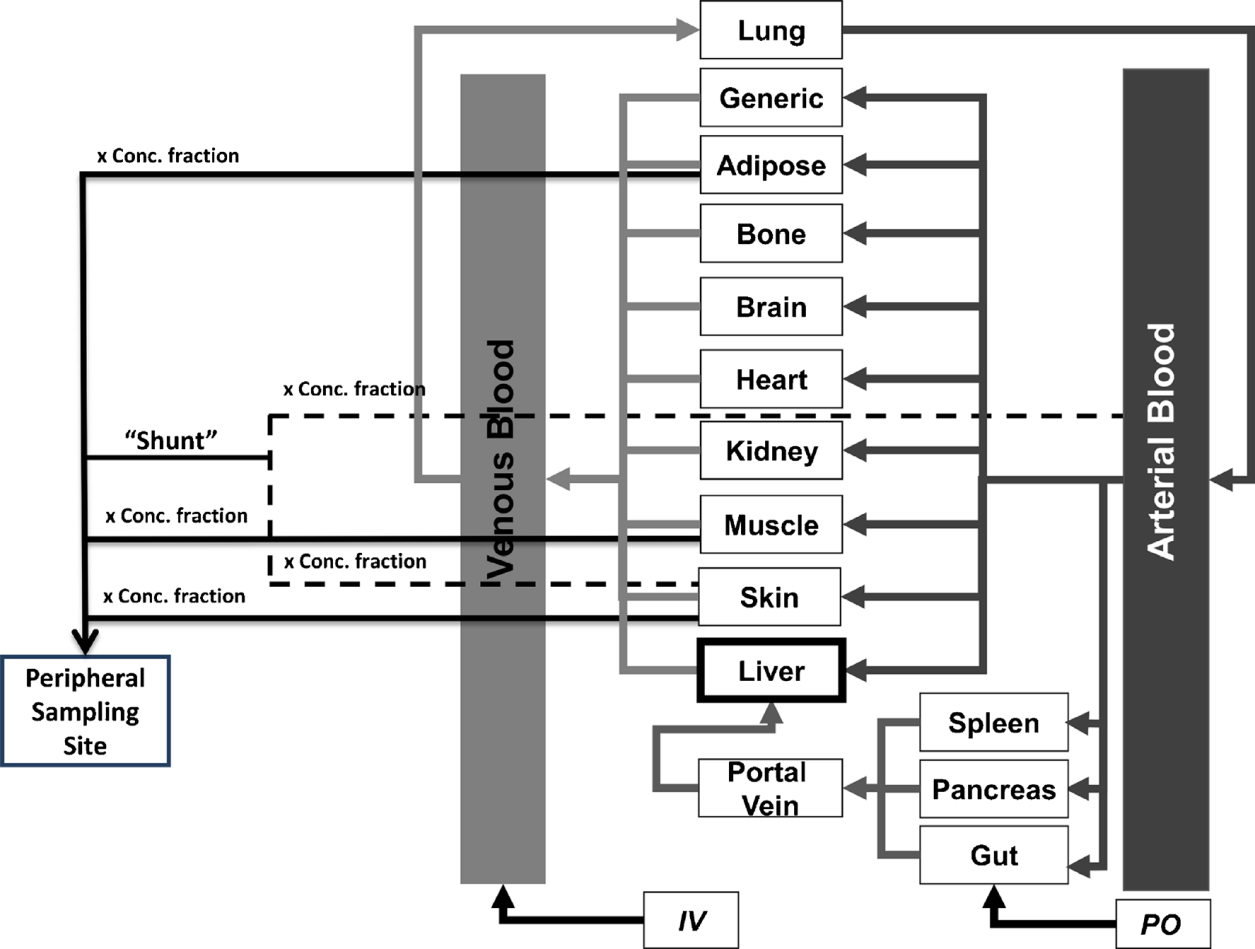
Current full-PBPK model in Simcyp with schematic representation of the peripheral sampling site model. The term *Conc. fraction* represents the fraction applied to each tissue concentration in the model

The tissue concentrations selected for incorporation in the model were skin, adipose, muscle and a “shunt” compartment. These tissues are logical selections since they can be found in the vicinity of the antecubital vein in the forearm, the most common clinical sampling site. The “shunt” compartment describes the arterio-venous anastomoses in the skin of the hand, where there is a direct connection of arterioles to the venules (18). The model published by Levitt includes these tissues; however, they describe the shunt as a new tissue and state “it is assumed the tissue supplied by the Shunt has the same pharmacokinetic properties as skin”, whereas for one of the models validated in this study a direct arterial contribution has been considered in combination with the skin contribution. In addition, the Levitt model also incorporates an “other” compartment, representing the subcutaneous space and connective tissue, which was not available in the Simulator model. Assuming the summation of all fractions in the model is equal to a total of 1; different options were explored to assess the implications of assigning this fraction to components available in the current PBPK model. A schematic of the model is shown in Fig. 1.

Four different model options were evaluated, three of which were variations of the fractions provided in the publication by Levitt (16). In the following equations, adipose/muscle/skin/lung conc refers to the tissue concentration observed in the simulation results and (*t*) refers to each time point where a predicted concentration has been output for the simulation. As the arterial concentration is not a standard output from the simulator, the lung concentration was converted, by dividing by Kp_Lung_/BP and used as a surrogate for the purposes of this exercise (Eq. 7). Kp is the tissue:plasma partition coefficient for the specified tissue which can be predicted using either the corrected Poulin and Theil (19–21), or Rodgers and Rowland (22–24) methods or measured data can be used directly. BP is the blood to plasma ratio required for the conversion of tissue concentration data to the concentration of drug exiting each tissue and entering the venous plasma at the peripheral sampling site.

A. Modified Levitt arm (LAC) model
  In this model, the fractions were based on those reported in the Levitt publication with the 10% assigned to ‘other’ being split equally between the skin, adipose, muscle and shunt:
  1 $$ \mathrm{Peripheral}\ \mathrm{Site}\ \mathrm{Conc}\mathrm{entration}\ (t)\ \mathrm{with}\ \mathrm{respect}\ \mathrm{t}\mathrm{o}\ \mathrm{plasma}=\left[0.1\cdot \frac{\mathrm{Adipose}\ \mathrm{Conc}(t)}{{}^{K{p}_{\mathrm{adipose}}}/{}_{\mathrm{BP}}}+0.075\cdot \frac{\mathrm{Muscle}\ \mathrm{Conc}(t)}{{}^{K{p}_{\mathrm{muscle}}}/{}_{\mathrm{BP}}}+0.275\cdot \frac{\mathrm{Skin}\ \mathrm{Conc}(t)}{{}^{K{p}_{\mathrm{skin}}}/{}_{\mathrm{BP}}}+0.55\cdot \mathrm{Shunt}\ \mathrm{Conc}(t)\right]\kern0.1em /\kern0.1em \mathrm{B}\mathrm{P} $$
  The Levitt model assigns the same distribution parameters and volume/blood flow ratio to the shunt as the skin and therefore the skin concentration was initially used as a surrogate for the shunt:
  2 $$ \mathrm{Shunt}\ \mathrm{Conc}(t)=\frac{\mathrm{Skin}\ \mathrm{Conc}(t)}{K{p}_{\mathrm{Skin}}\kern0.1em /\kern0.1em \mathrm{B}\mathrm{P}} $$
B. Modified Levitt “skin” (LAS) model
  In this instance, the 10% of the arm concentration assigned to ‘other’ by Levitt was assigned completely to the shunt (as skin concentration):
  3 $$ \mathrm{Peripheral}\ \mathrm{Site}\ \mathrm{Conc}\mathrm{entration}\ (t)\ \mathrm{with}\ \mathrm{respect}\ \mathrm{t}\mathrm{o}\ \mathrm{plasma}=\left[0.075\cdot \frac{\mathrm{Adipose}\ \mathrm{Conc}(t)}{{}^{K{p}_{\mathrm{adipose}}}/{}_{\mathrm{BP}}}+0.05\cdot \frac{\mathrm{Muscle}\ \mathrm{Conc}(t)}{{}^{K{p}_{\mathrm{muscle}}}/{}_{\mathrm{BP}}}+0.25\cdot \frac{\mathrm{Skin}\ \mathrm{Conc}(t)}{{}^{K{p}_{\mathrm{skin}}}/{}_{\mathrm{BP}}}+0.625\cdot \mathrm{Shunt}\ \mathrm{Conc}(t)\right]\kern0.1em /\kern0.1em \mathrm{B}\mathrm{P} $$
  The shunt equation is the same in this model as described in the LAC model.
C. Modified Levitt arm “arterial” (LAA) model
  The lack of arterial contribution in the shunt for the LAC and LAS model was not considered to be physiologically representative, so in the third model, the fraction for “other” was assigned to the shunt but used arterial concentration, as shown in Eq. 6. This 10% was removed from the skin fraction that was representing the shunt in the LAS model.
D. Physiological arm concentration (PAC) model
  Literature searches were used to identify relevant physiological data that could describe the tissue concentration fractions required for this model. These fractions were derived from reported relative blood flows in the forearm (25–29). Unfortunately, these data are limited and no description of the contribution of the anastomoses to the antecubital vein blood flow could be obtained. The described fractions for this model therefore only refer to the adipose, muscle and skin concentrations:
  4 $$ \mathrm{Peripheral}\ \mathrm{Site}\ \mathrm{Conc}\mathrm{entration}\ (t)\ \mathrm{with}\ \mathrm{respect}\ \mathrm{t}\mathrm{o}\ \mathrm{plasma}=\left[0.1\cdot \frac{\mathrm{Adipose}\ \mathrm{Conc}(t)}{{}^{K{p}_{\mathrm{adipose}}}/{}_{\mathrm{BP}}}+0.6\cdot \frac{\mathrm{Muscle}\ \mathrm{Conc}(t)}{{}^{K{p}_{\mathrm{muscle}}}/{}_{\mathrm{BP}}}+0.3\cdot \frac{\mathrm{Skin}\ \mathrm{Conc}(t)}{{}^{K{p}_{\mathrm{skin}}}/{}_{\mathrm{BP}}}\right]\kern0.1em /\kern0.1em \mathrm{B}\mathrm{P} $$
  Since the sampling volume is usually small in relation to the total blood volume, it was assumed to be negligible in this case, and therefore mass balance is maintained in the underlying PBPK model. Calculations of the peripheral site profile were performed in Microsoft Excel 2010 (Microsoft Corporation, Redmond, WA, USA) using the mean concentration-time profiles for each tissue.
  Following the initial validation steps of these models, the arterial contributing fraction in the LAA was varied from 0.02 to 0.625, with skin fraction in the shunt being increased/decreased to maintain a total shunt fraction of 0.625, to assess the impact on C_max_ prediction and potentially further refine the model.

### Compound Data and Simulations

Literature searches for drugs with an available Simcyp Simulator compound dataset were conducted to identify clinical studies where the dose was administered by i.v. infusion, and *in vivo* concentration-time data were available. Data extracted from the publications included the reported steady-state volume of distribution (Vss), in addition to the *in vivo* concentration-time data and study details. Where concentration-time data were only presented in figures, the data were extracted using the GetData graph digitiser version 2.22 (Get Data Graph Digitizer, 2012, http://getdata-graph-digitizer.com/) for comparative purposes. Where Vss was not reported for a specific study, this was obtained by non-compartmental analysis using Phoenix version 1.3 (Pharsight), utilising the extracted concentration-time profile data. Physicochemical and elimination data already present in the Simcyp compound file were utilised.

It was assumed that reasonable prediction of Vss can be used as an indication of the correctness of the predicted Kp values used in the PBPK model. However, clearly this is necessary but may not guarantee that all Kp values are correct. Since Vss is a description of the distribution under steady-state conditions, it should theoretically be unaffected by early distribution differences. For the purposes of these simulations, Kp values and the overall Vss were initially predicted using “Method 2” within the simulator. In some cases, the predicted Vss did not match the observed values; therefore, all tissue Kp values were scaled up or down to recover the observed Vss prior to the simulations being performed. Suitable values for the universal Kp scalar were determined using the inbuilt sensitivity analysis or parameter estimation tools within the platform.

Simulations were performed using the Simcyp Simulator (Version 13 release 1) (Simcyp, Sheffield, UK), with the provided healthy volunteer population and concentration time points equally divided over 2000 time points for the length of the simulation period. Study design and dosing regimen were matched to the published trial design for all studies.

### Analysis and Comparisons

Visual comparisons of reported *in vivo* and predicted concentration-time profiles were made. Predicted C_max_ values were compared with observed values. As the time point of the highest concentration (T_max_) in simulations can differ from the reported T_max_, predicted and observed concentrations were compared at the *in vivo* T_max_. This is due to the fact that the simulation reports 2000 time points whereas in clinical studies, a finite number of samples can be taken. The fold difference between these observed and predicted values for C_max_ was calculated as a measure of prediction accuracy, with the number of results between 0.8- and 1.25-fold or within ±2-fold recorded. Predicted C_max_ values within 2-fold of reported *in vivo* values were considered to be reasonable predictions. The prediction accuracy of all the models described above was compared for selection of the most suitable peripheral site model.

## RESULTS

### Compound Data

A total of 15 studies with i.v. administration *via* infusion were identified from the published literature, covering seven compounds, where the relevant compound data were available in the Simcyp Simulator. The compounds investigated were alprazolam, imipramine, metoprolol, midazolam, omeprazole, rosiglitazone and theophylline (*n* = 4, 3, 1, 2, 2, 1 and 2 sets of observed data, respectively). The studies investigated are summarised in Table I.

**Table I:** Summary of Studies Identified for Validation

Drug	Infusion length (min)	First sampling time (min)	LogP	Observed Vss (L/kg)	Predicted Vss^*b*^ (L/kg)	Dose	Study reference	Study identifier
Alprazolam	2	16	2.12	0.57	1.56	0.5 mg	Kroboth 1988 (40)	1
	2	20		0.55		2 mg	Kroboth 1988 (40)	2
	1	12		0.86^*a*^		0.5 mg	Lin 1988 (35)	3
	30	30		1.14^*a*^		1 mg	Venkatakrishnan 2005 (41)	4
Imipramine	30	68	4.8	23.6^*a*^	8.19	12.5 mg	Abernethy 1984 (42)	5
	30	65		19.8		12.5 mg	Abernethy 1984 (43)	6
	30	30		17.3^a^		12.5 mg	Ciraulo 1988 (44)	7
Metoprolol	5	5	1.88	3.68^*a*^	3.12	11.5 mg	Richard 1994 (45)	8
Midazolam	2	15	3.53	2.1	4.56	0.05 mg/kg	Palkama 1999 (46)	9
	2	43		1.2		0.05 mg/kg	Saari 2006 (47)	10
Omeprazole	5	5	2.23	0.5	0.36	40 mg	Oosterhuis 1992 (48)	11
	5	5		0.43		80 mg	Oosterhuis 1992 (48)	12
Rosiglitazone	60	60	2.6	0.19	0.09	2 mg	Cox 2000 (34)	13
Theophylline	20	20	−0.02	0.47	0.31	5 mg/kg	Aslaksen 1981 (49)	14
	5	10		0.33		197 mg	Jackson 1986 (50)	15

^*a*^Obtained from extracted concentration-time data by non-compartmental analysis using Phoenix

^*b*^Prior to correction with a Kp scalar

*Vss* steady-state volume of distribution

### Comparison of Models

**Fig. 2:**
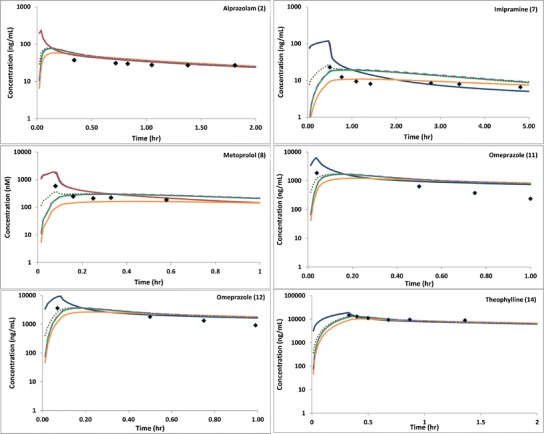
Observed and predicted concentration-time profiles for all models studied for a selection of studies. *Black* diamonds represent observed *in vivo* data. *Red* and *blue lines* represent the predicted arterial and central venous profiles, respectively. The tested models are represented by *green line* (LAC), *yellow line* (PAC), *purple dash* (LAS) and *green dots* (LAA)

**Fig. 3:**
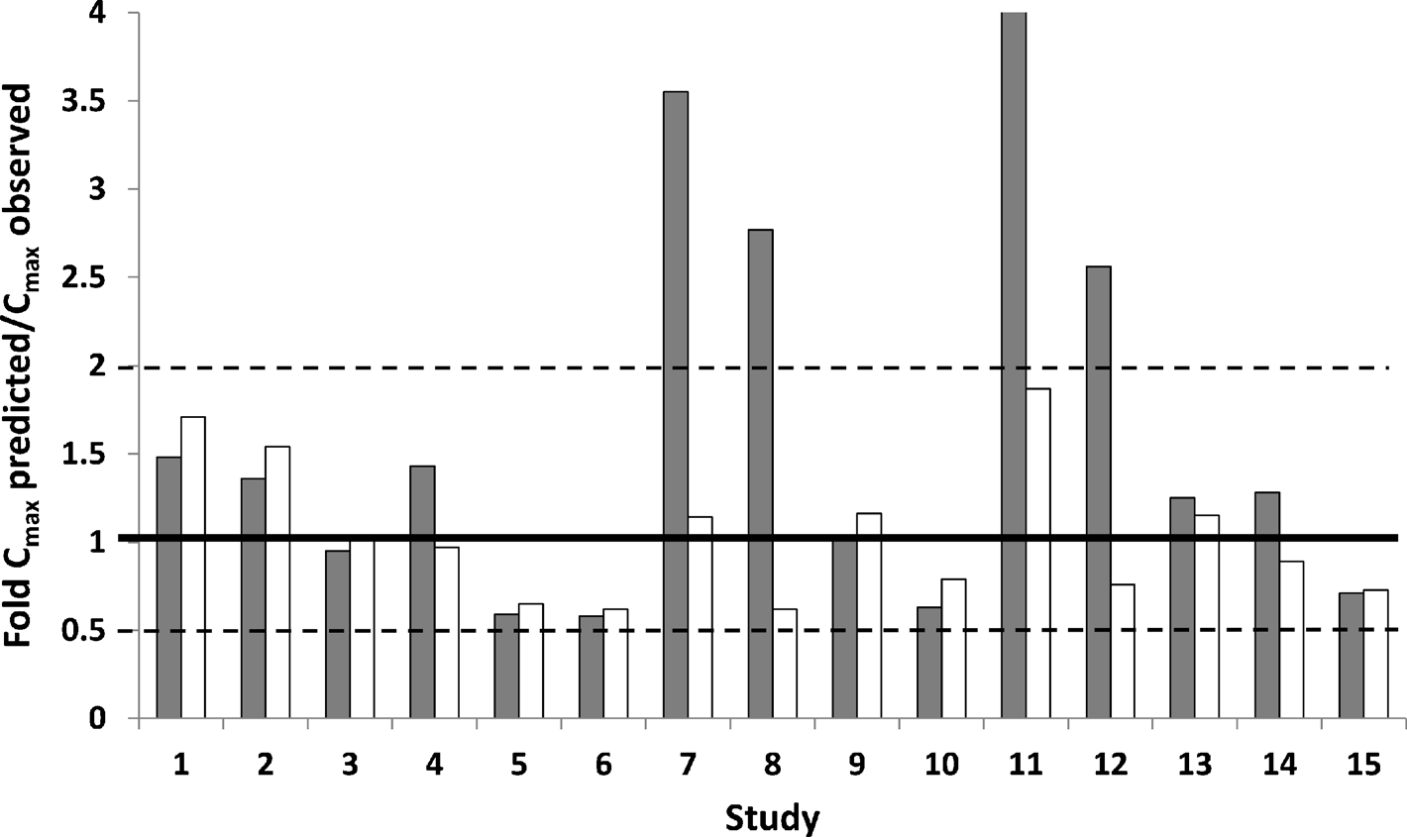
Fold differences between predicted/observed C_max_ concentration for the central venous (*grey bars*) and LAA (*white bars*) models. *Solid line* represents the line of unity; dashed lines represent a 2-fold difference from observed values

A visual comparison was made of the early-stage concentration-time profiles from published studies with predicted profiles using the different models described above. Selected key profiles are shown in Fig. 2, with results for all studies available in the supplementary material. Clear improvements in predictions can be seen for a number of studies, metoprolol and omeprazole in particular, with the LAA simulated C_max_ generally closest to the observed C_max_. However, it can be noted from the profiles (see supplementary material) that in a number of cases, the simulated profiles for all of the models have converged at an earlier time point than an observation has been made in the *in vivo* studies. This is true even when a difference was predicted between the initial phase of the peripheral sampling site and central venous profiles and makes validation of the models difficult. The fold difference between the observed and predicted concentrations at the observed T_max_ for all models is shown in Table II, with summary statistics in Table III and a graphical summary of the prediction accuracy for the LAA is given in Fig. 3. The LAA is considered to be the most accurate model, with all of the predicted concentrations within 2-fold of the observed values. This comparison confirms the utility of the corrective model, particularly for those compounds identified previously where early sampling time points are available. The final model selected based on these results was the LAA, which incorporates the arterial fraction in the shunt.

**Table II:** Results from Model Comparison

Drug	Study	Observed C_max_^*a*^ (ng/mL)	Simulated concentration at observed T_max_						Simulated/observed concentration at observed T_max_					
			Venous plasma	Arterial conc	LAC	LAS	LAA	PAC	Venous plasma	Arterial conc	LAC	LAS	LAA	PAC
Alprazolam	1	9.48	14.0	14.2	16.1	16.5	16.2	14.3	1.48	1.50	1.70	1.74	1.71	1.51
	2	37.0	50.5	51.0	56.8	57.9	56.9	53.2	1.36	1.38	1.54	1.56	1.54	1.44
	3	12.6	12.0	12.0	13.0	13.4	13.1	9.97	0.95	0.95	1.03	1.06	1.04	0.79
	4	19.7	28.2	28.7	17.6	18.2	19.1	13.3	1.43	1.46	0.89	0.92	0.97	0.68
Imipramine	5	31.6	18.7	18.8	19.9	20.8	20.4	10.5	0.59	0.59	0.63	0.66	0.65	0.33
	6	32.0	18.6	18.7	19.2	20.1	19.7	10.5	0.58	0.58	0.60	0.63	0.62	0.33
	7	22.8	80.9	99.8	17.3	18.1	26.1	9.39	3.55	4.38	0.76	0.79	1.14	0.41
Metoprolol^*b*^	8	585	1623	1843	195	202	364	98.7	2.77	3.15	0.33	0.35	0.62	0.17
Midazolam	9	46.7	47.7	47.8	53.6	55.8	54.4	36.9	1.02	1.02	1.15	1.19	1.16	0.79
	10	59.2	37.3	37.6	47.5	48.3	47.0	48.1	0.63	0.64	0.80	0.82	0.79	0.81
Omeprazole	11	629	6306	6344	587	610	1177	374	10.0	10.1	0.93	0.97	1.87	0.59
	12	3582	9173	9255	1959	2035	2738	1282	2.56	2.58	0.55	0.57	0.76	0.36
Rosiglitazone	13	115	144	146	130	131	132	121	1.25	1.27	1.13	1.14	1.15	1.05
Theophylline	14	14,602	18,747	19,089	12,141	12,411	13,013	8932	1.28	1.31	0.83	0.85	0.89	0.61
	15	14,543	10,361	10,458	10,461	10,693	10,614	7446	0.71	0.72	0.72	0.74	0.73	0.51

^*a*^Highest observed concentration

^*b*^Kp scalar fitting not used. Metoprolol concentration data is given in nM

*LAC* Modified Levitt arm model, *LAS* Modified Levitt “skin” model, *LAA* Modified Levitt arm “arterial” model, *PAC* physiological arm concentration model

**Table III:** Summary Statistics for Evaluated Models

	Venous plasma	Arterial Conc	LAC	LAS	LAA	PAC
Average simulated C_max_/observed C_max_	2.01	2.11	0.91	0.93	1.04	0.69
*N* within 0.8–1.25 observed	2	2	6	7	6	2
*N* > 2-fold different to observed	4	4	1	1	0	5

Total number of datasets evaluated for all models = 15

*LAC* Modified Levitt arm model, *LAS* Modified Levitt “skin” model, *LAA* Modified Levitt arm “arterial” model, *PAC* physiological arm concentration model

### Systematic Alteration of the Arterial Contribution

**Fig. 4:**
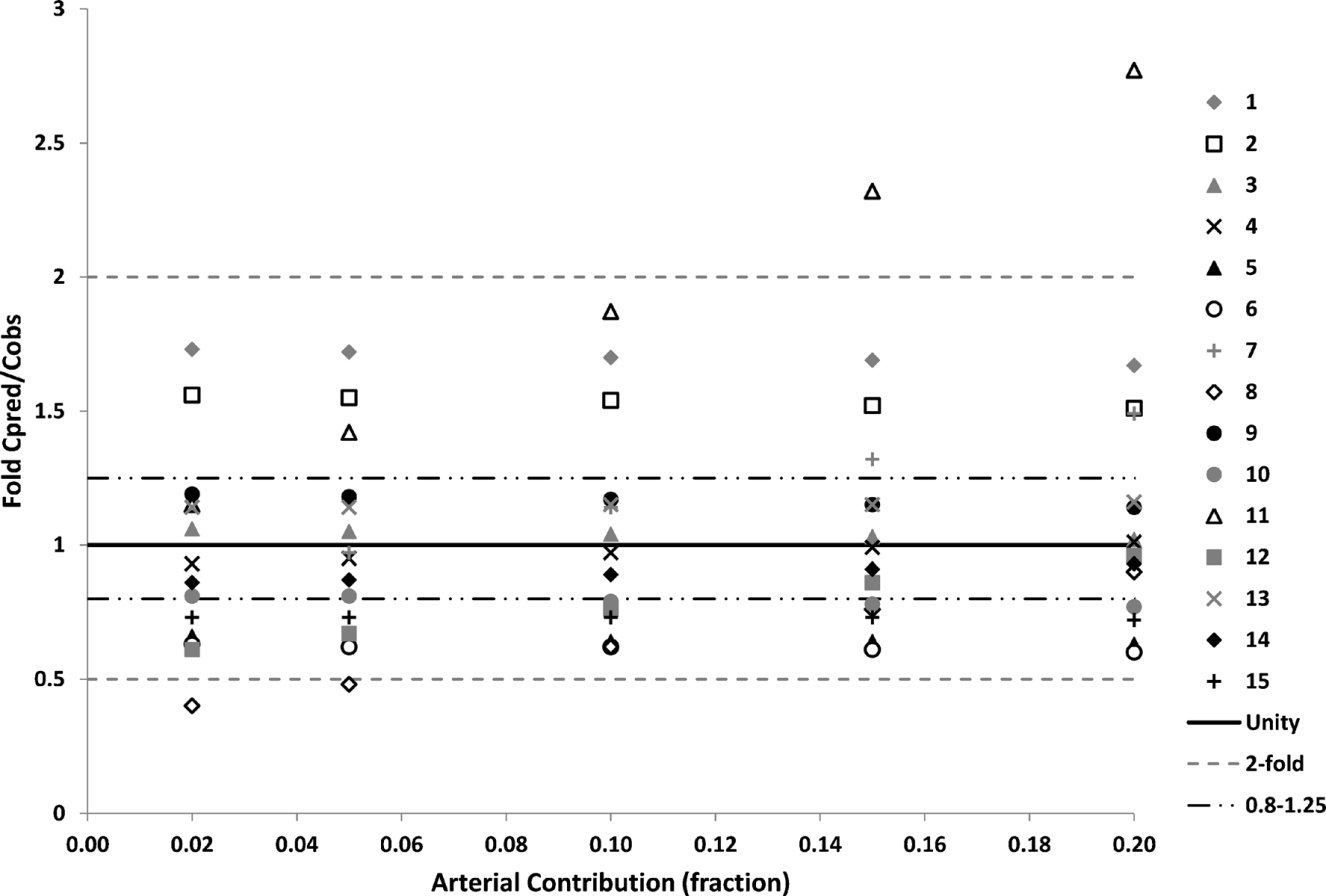
Effect of varying the arterial contribution to the peripheral sampling site concentration on fold difference in predicted and observed C_max_ using the LAA model. Individual markers represent each study investigated; *solid line* represents the line of unity; *dot-dash lines* represent 0.8- to 1.25-fold difference from observed values; *dashed lines* represent a 2-fold difference from observed values

Changing the fractional contribution of the arterial concentration to the shunt resulted in an improvement for some compounds, bringing the C_max_ predictions within a 0.8- to 1.25-fold range, while other compounds moved outside the 2-fold range (Table IV). A C_max_ prediction within 2-fold of the *in vivo* value was observed for all studies when using the LAA with a fraction of 0.1 for the arterial contribution. Figure 4 gives a graphical representation of the consequences of changing this fraction, though due to extremely poor results, the arterial fraction of 0.625 is excluded from this figure. These observations suggest that the original fraction of 0.1 for arterial contribution was preferable and should be maintained in the final model. However, further validation with a larger observed dataset is required.

**Table IV:** Results from Changing the Arterial Fraction

Drug	Study	Simulated/observed concentration at observed T_max_						
		Venous plasma	Arterial contribution to peripheral sampling site concentration					
			0.02	0.05	0.1	0.15	0.2	0.625
Alprazolam	1	1.48	1.73	1.72	1.70	1.69	1.67	1.52
	2	1.36	1.56	1.55	1.54	1.52	1.51	1.40
	3	0.95	1.06	1.05	1.04	1.03	1.02	0.94
	4	1.43	0.93	0.95	0.97	0.99	1.01	1.21
Imipramine	5	0.59	0.66	0.65	0.64	0.64	0.63	0.57
	6	0.58	0.63	0.62	0.62	0.61	0.60	0.56
	7	3.55	0.86	0.97	1.14	1.32	1.49	2.98
Metoprolol	8	2.78	0.40	0.48	0.62	0.76	0.90	2.08
Midazolam	9	1.02	1.19	1.18	1.17	1.15	1.14	1.01
	10	0.63	0.81	0.81	0.79	0.78	0.77	0.68
Omeprazole	11	10.0	1.15	1.42	1.87	2.32	2.77	6.61
	12	2.56	0.61	0.67	0.76	0.86	0.96	1.79
Rosiglitazone	13	1.25	1.14	1.14	1.15	1.15	1.16	1.21
Theophylline	14	1.28	0.86	0.87	0.89	0.91	0.93	1.11
	15	0.71	0.73	0.73	0.73	0.73	0.72	0.70
Average simulated C_max_/obs C_max_		2.01	0.95	0.99	1.04	1.10	1.15	1.62
*N* within 0.8–1.25 observed		3	8	7	6	6	7	5
*N* > 2-fold different to observed		4	1	1	0	1	1	3

Total number of datasets evaluated for all models = 15

The final selected fractions for use in the LAA are shown in Table V, and the final equations are shown below.

5 $$ \mathrm{Peripheral}\ \mathrm{Site}\ \mathrm{Con}\mathrm{c}\mathrm{entration}\ (t)\ \mathrm{with}\ \mathrm{respect}\ \mathrm{t}\mathrm{o}\ \mathrm{plasma}=\left[0.075\cdot \frac{\mathrm{Adipose}\ \mathrm{Conc}(t)}{{}^{K{p}_{\mathrm{adipose}}}/{}_{\mathrm{BP}}}+0.05\cdot \frac{\mathrm{Muscle}\ \mathrm{Conc}(t)}{{}^{K{p}_{\mathrm{muscle}}}/{}_{\mathrm{BP}}}+0.25\cdot \frac{\mathrm{Skin}\ \mathrm{Conc}(t)}{{}^{K{p}_{\mathrm{Skin}}}/{}_{\mathrm{BP}}}+\mathrm{Shunt}\ \mathrm{Conc}(t)\right]\kern0.1em /\kern0.1em \mathrm{B}\mathrm{P} $$

6 $$ \mathrm{Shunt}\ \mathrm{Conc}(t)=0.525\cdot \frac{\mathrm{Skin}\ \mathrm{Conc}(t)}{{}^{K{p}_{\mathrm{skin}}}/{}_{\mathrm{BP}}}+0.1\cdot \mathrm{Arterial}\ \mathrm{Conc}(t) $$

7 $$ \mathrm{Arterial}\ \mathrm{Conc}(t)=\frac{\mathrm{Lung}\ \mathrm{Conc}(t)}{{}^{K{p}_{\mathrm{lung}}}/{}_{\mathrm{BP}}} $$

**Table V:** Final Selected Fractions

Tissue	Concentration fraction
Adipose	0.075
Skin	0.25
Muscle	0.05
Shunt-skin	0.525
Shunt-arterial	0.1

## DISCUSSION

A systematic validation and comparison of a number of methods to improve the prediction of C_max_ from PBPK models has been performed and reported. Models that were evaluated were based on the full PBPK model in the Simcyp Simulator (Version 13 Release 1) and include standard tissue concentration outputs to describe the concentration-time profile for venous plasma at a peripheral sampling site, *i.e.* the antecubital vein. While the consideration was made at the inception of this model that construction of a PBPK model or compartment for arm might be a possible solution, the model selected allows the use of the existing physiologically based model as implemented in the Simulator and the associated outputs. This model provides an elegant solution where mass balance is maintained in the original model, and comparisons can be easily drawn between the concentrations in the central venous compartment and at the new peripheral sampling site. It is important to note that all of the validation is performed based on peripheral sampling data. The perfect scenario to validate this model would be the use of both peripheral and central venous or arterial sampling data for comparative purposes; however, such data are experimentally difficult and ethically questionable to obtain in humans.

Based on the current validation, the LAA model was considered to give the best performance of all of the models investigated, with the predicted C_max_ closest to the observed values, and within the specified 2-fold range for all studies. This 2-fold range is a commonly used criterion for assessing the accuracy of predictions in *in vitro–in vivo* extrapolation and has recently been subject to some debate, with additional models for success criteria being proposed, though there is no consensus on the most appropriate method for validation (30). The LAA model is also considered to be the most physiologically accurate, where the shunt considers a contribution from the arterial blood in line with the role of the arterio-venous anastomoses in the skin of the hand, this is consistent with descriptions of the circulatory physiology of the skin (18). This contributing factor is absent from all other models investigated in this study (LAC, LAS and PAC). The lack of physiological data, particularly relating to the shunt, that could be identified in the literature when building the alternative PAC model is disappointing. This factor potentially contributes to the poor performance of this model in comparison to the LAA. The final variations of the arterial fractions made some improvement to the selected model for specific compounds, but led to a worsening prediction for others. Overall, it was considered that a model giving a reasonable prediction for all studies was preferred to improving the prediction of one or two compounds at the expense of accuracy for others, potentially skewing the model. Usually, over-fitting of a model for one or two cases can reduce its generalisation ability. In addition, the final value selected is in line with observed data cited by Chiou (7) where arterio-venous shunting in dog leg is observed to be approximately 4% of blood flow, which gives confidence that the final value of 10% is physiologically reasonable in the absence of any observed human data.

An assumption of the current approach is that accurate prediction of Vss means the Kp values are correct, so a drawback to the model is that it relies on the accurate prediction of Vss values within the underlying PBPK model. On this basis and using the *in vivo* Vss values, a fitting strategy was used to ensure the distribution volume was reasonably described (by an accurate Vss prediction) before proceeding to predict the peripheral sampling site concentration. Scaling of Kp values was required for all compounds apart from metoprolol. However, if *in vivo* data are lacking to validate the Vss or Kp values or if a bottom-up approach is intended, the confidence in the prediction of distribution cannot be validated. If animal data are available, these can be utilised within the model to ensure the distribution is reasonably described or the physicochemical properties of the compound can be used to guide the selection of a predictive model ((31–33), personal communication from Iain Gardner).

In the initial stages of this project, the use of different fractions in the models for compounds with different Vss values or physicochemical parameters was considered. This was ultimately dismissed, as the reason for the differences in the central venous and the peripheral sampling site concentrations depends on both the compound and physiological parameters, and therefore, the model structure should not be compound dependent. However, compound-specific differences may be evident in the concentration-time profiles, as lipophilic drugs will be expected to distribute more extensively into tissues leading to bigger site-specific concentration differences at early times after drug administration (7). These compound-specific differences are already incorporated in the underlying PBPK model, with lipophilicity and protein-binding considered as contributing factors for the prediction of Kp values. The resultant differences can be observed in the profiles provided in Fig. 2 and in the supplementary material, *e.g.* theophylline with low logP and Vss values has minimal differences between the concentration profiles for the central venous compartment and the peripheral sampling site model, whereas imipramine with both high Vss and logP shows significant differences. For other compounds, the contributions of these factors are less clear. The lack of reported early time points in the observed data for these compounds limits the conclusions that can be drawn as the convergence of predicted concentration profiles prior to the observed data hinders the determination of which model fits best in these circumstances. It is worth noting that omeprazole does not have a particularly high logP or Vss, and significant differences are noted in the profiles—suggesting other factors may also be contributing to this phenomenon (*e.g.* charge/compound type). With such a limited clinical dataset, it is challenging to explore the potential causes further and draw any strong conclusions. It should also be noted that no consideration was given to the accuracy of clearance values in this study, and the elimination phase of each simulated profile was not compared to the *in vivo* situation. The assumption is made that for low and moderate clearance compounds, there will be little impact on the C_max_ value, meaning the simulated results remain valid for all of the compounds that were investigated in this study.

The selection of the predicted concentration at the time point of the highest observed concentration for the calculation of fold differences is a key factor in understanding the results. Comparisons can be drawn between the highest observed and predicted concentrations, but these could be potentially meaningless as the T_max_ reported from the simulation can differ substantially from that observed. Over the course of each simulation in this study, 2000 data points are reported whereas the clinical/observed data is usually limited to around 10–20 samples over several hours. This can be particularly problematic for comparisons when the first sampling time point is much later than the end of infusion and/or the T_max_ observed in the simulation, as there is no way of knowing what is happening *in vivo* where there are no reported observations. While all compounds showed an improvement in terms of fold error in C_max_ prediction when using the LAA model, it is obvious from the comparison of the observed and simulated concentration-time profiles that the utility of the model can only be fully tested when concentration data for early sampling time points are available. This is in agreement with Chiou (7) and Shankaran (1) who highlighted the differences occurring in the initial phases of the concentration-time profiles. These time points are expected to be within the initial distribution phase (*e.g.* 5–10 min after administration), and samples taken at >30 min after administration are too late to observe the expected differences in the central venous and peripheral sampling site concentrations. Comparing the simulated profiles to the *in vivo* data (Fig. 2), it seems that only three studies, for metoprolol and omeprazole, had early enough time points to fully illustrate the benefit of using the corrective model and in most of these cases, only one point is relevant. As can be seen in Fig. 2 and also in the supplementary material, most studies have clearly reached an equilibration point before the first *in vivo* sampling time and the predicted profiles have converged. The differences between the models are mainly limited to the initial distribution phase, which is in line with the observations made by others in this arena (1, 7). A few exceptions arise in this dataset; in study 4 for alprazolam and study 7 for imipramine, the first sampling point is not until 30 min after infusion, yet a significant correction using the peripheral sampling site model was still observed at this time, suggesting that in some cases, distribution differences can persist for longer than the initial 5–10 min.

Another consideration is variability, as the model has been compared only to the highest concentration point extracted digitally from the mean concentration-time profile for each dataset. C_max_ may not be at the same time for all individuals, hence biasing the C_max_ of the mean concentration profile. Out of the 15 datasets studied, only two (34, 35) had tabulated C_max_ and T_max_ data for the i.v. study with a measure of variability (range or standard deviation) available. Variability in the reported C_max_ values was <30%, and the reported mean C_max_ (taken as the mean of individual C_max_ values) was similar to the C_max_ value determined from the mean concentration profile (12.6 *vs* 12.6 ng/mL and 146 *vs* 115 ng/mL for alprazolam and rosiglitazone, respectively). In addition, the numbers of subjects considered in the studies are relatively low (*n* values range from 1 to 14). Both of these issues could lead to uncertainty in the validation of the model. This is due to a paucity of data; in an ideal scenario, individual data for a large number of subjects would be available, ensuring that variability within the wider population is fully captured with best- and worst-case scenarios fully represented. This is a general problem with validating models and does not only apply to this study, but it becomes more pertinent when dealing with such a limited number of datasets. The Simulator reports concentration-time points for each simulated individual within a virtual trial for plasma and individual tissues, so if clinical data were available, further validation in this area would be feasible.

Expansion of the underlying models could also be considered in the future to include the effect of certain drugs on the cardiovascular system or the impact of the application of heat on the distribution at the sampling site. An example of this is metoprolol, which alters heart rate, cardiac output and consequently tissue blood flows (36, 37). If metoprolol distribution is perfusion-limited then it could be affected by the action of the drug on the blood flows. Similarly, the arterial contribution could be increased to mimic the effect of a temperature increase in the vicinity of the sampling site. While in the current study, it is assumed, in the absence of further information, that the conditions of a standard temperature and a sedentary subject are met, the use of a heat-box to induce an “arterialised concentration” at a venous site have been documented previously (38, 39). However, the development of such models is outside the scope of the current study.

The initial constraints of this investigation include the availability of both a library compound within the Simulator and an *in vivo* study utilising an infusion administration, the validation was therefore limited by the sampling points in those studies. Further i.v. infusion studies with relevant sampling time points have been identified for compounds not currently available in the Simcyp Simulator. This extended validation requires the development of additional compound files which was outside the scope of this initial study. As proof of concept, the improvement shown in this report seems to be adequate and allows a basis for further validation as well as investigation into potential applications within a number of different arenas.

## CONCLUSION

A peripheral sampling site model that improves the prediction of C_max_ values has been developed and validated in this study. The tissue fractions that constitute the sampling site concentration were tested for seven compounds and provide a good concordance between the observed and predicted C_max_ values. Further validation is required with more compounds and clinical studies, particularly those with early sampling time points, to confirm the utility of the model. However, the potential for the use of such a model has been adequately illustrated herein. It is envisaged these models can be enrolled as built-up modules within PBPK platforms to address potential affections and variability of the initial mixing of the blood at the site of sampling (such as effect of heat, variation in adipose and muscle content of the body), and hence, they may give a more realistic simulated drug concentrations in blood or plasma at early time points.

## Electronic supplementary material

ESM 1 (PDF 272 kb)
