# Supplementary material for: Are Physiologically Based Pharmacokinetic Models Reporting the Right Cmax? Central Venous Versus Peripheral Sampling Site
Source: AAPS J. 2015 Jun 23;17(5):1268–79. doi: 10.1208/s12248-015-9796-7 (PMC4540731; doi:10.1208/s12248-015-9796-7)

Supplementary material

Observed and predicted concentration-time profiles for all models studied. Black diamonds represent observed in vivo data. Red and Blue lines represent the predicted Arterial and Central Venous profiles respectively. The tested models are represented by: Green line (LAC), Yellow line (PAC), Purple dash (LAS) and Green dots (LAA)

Alprazolam

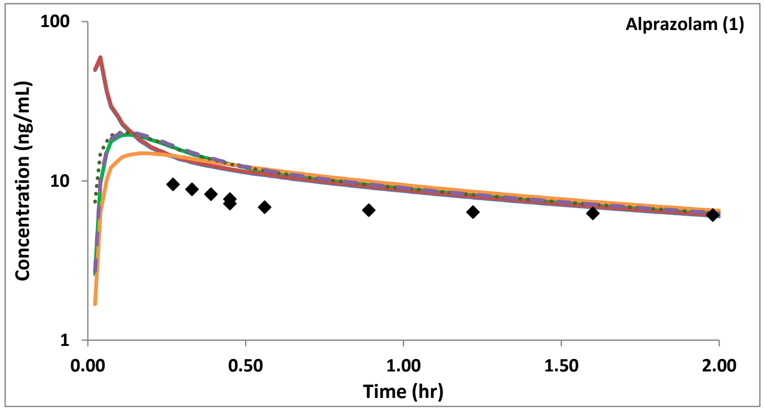


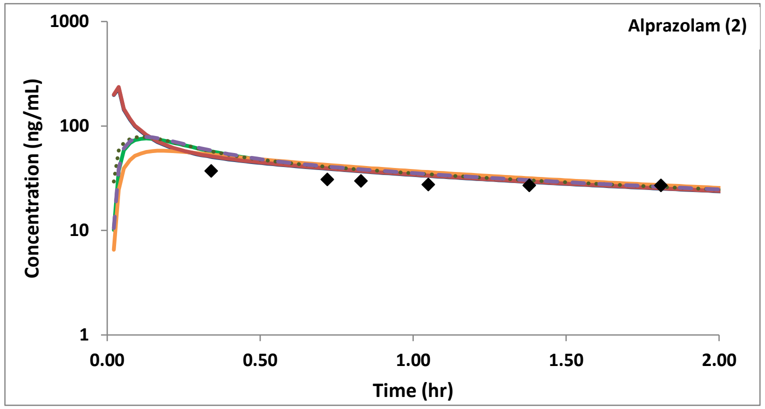


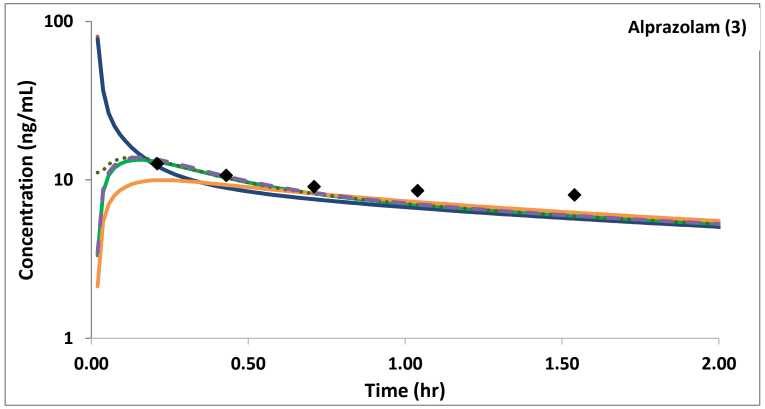


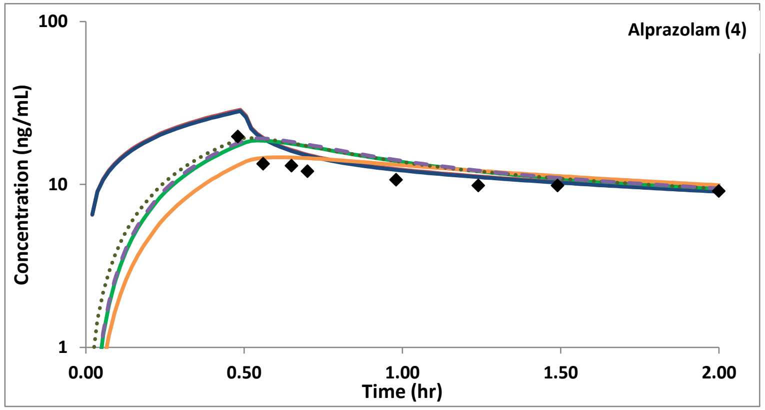

Imipramine

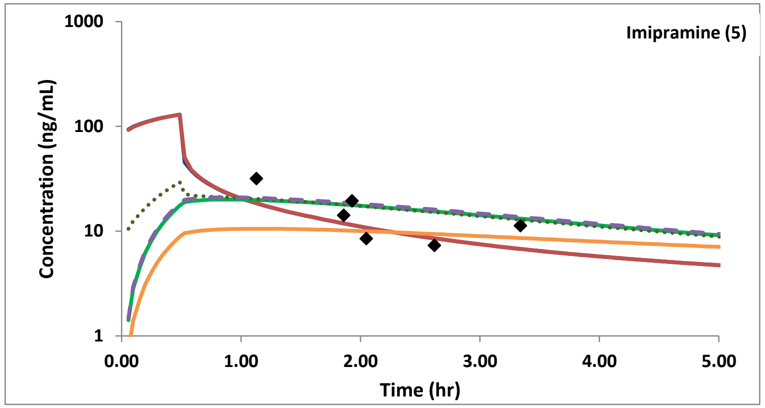
Imipramine (5)
1000
100
10
1
Concentration (ng/mL)
0.00
1.00
2.00
3.00
4.00
5.00
Time (hr)

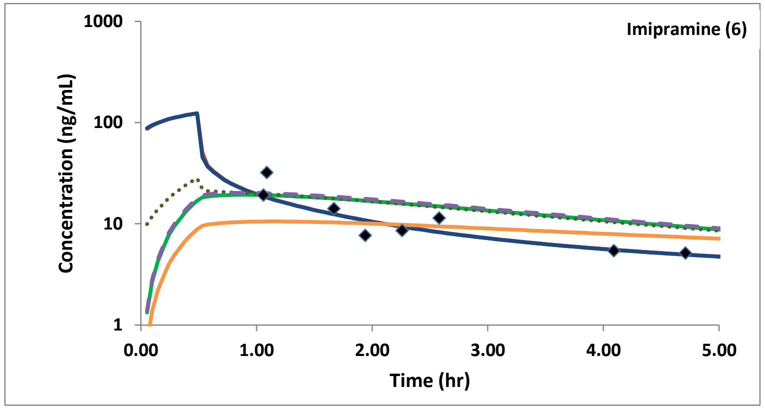
Imipramine (6)
1000
100
10
1
Concentration (ng/mL)
0.00
1.00
2.00
3.00
4.00
5.00
Time (hr)

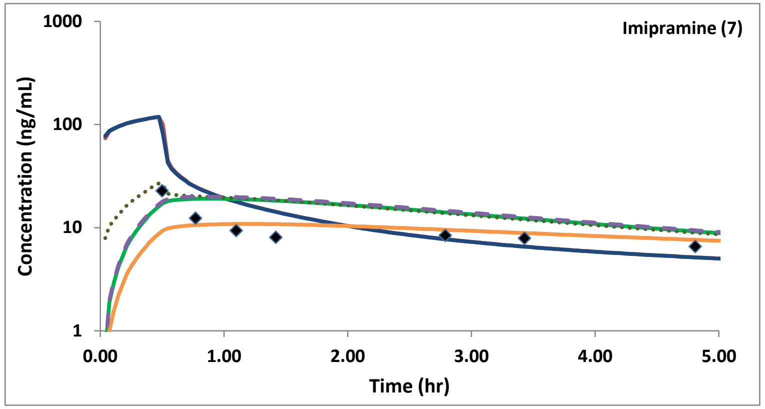
Imipramine (7)
1000
100
10
1
Concentration (ng/mL)
0.00
1.00
2.00
3.00
4.00
5.00
Time (hr)

Metoprolol

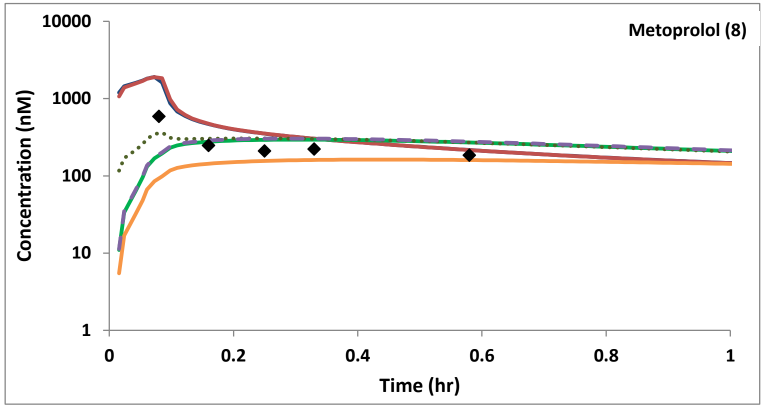


Midazolam

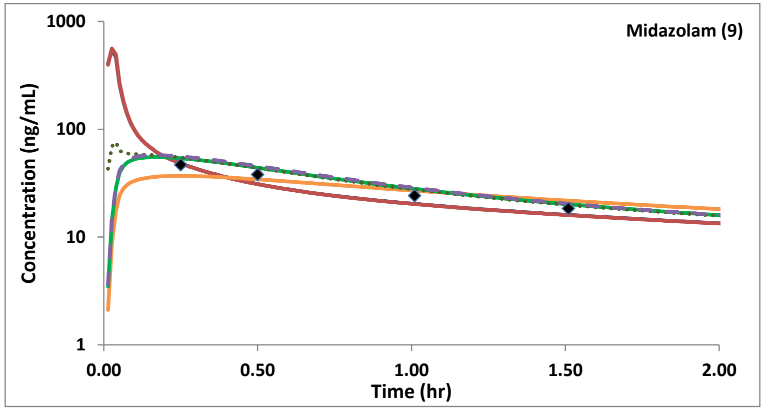


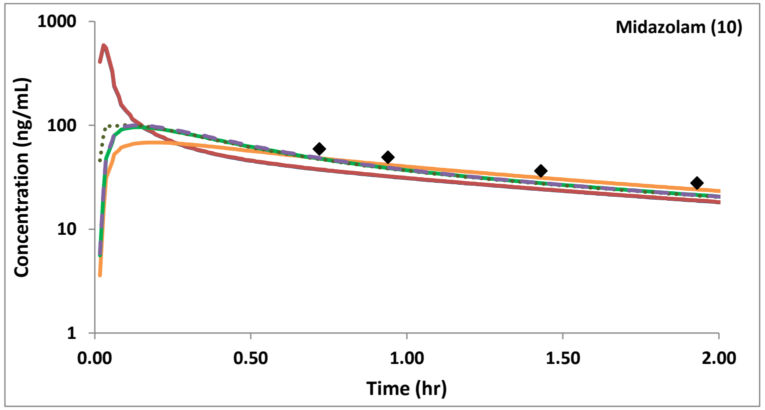

Omeprazole

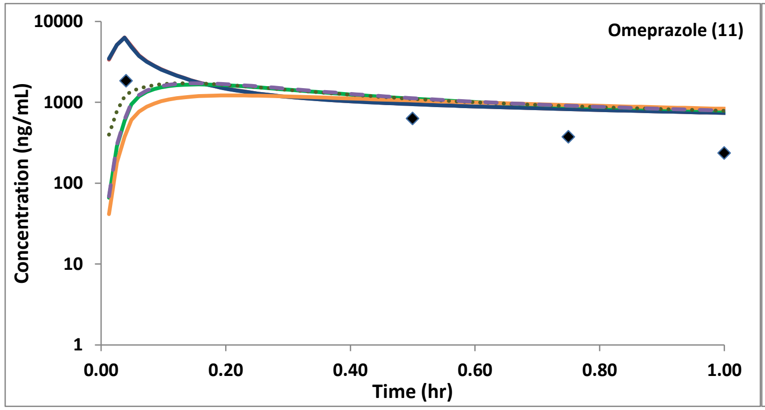


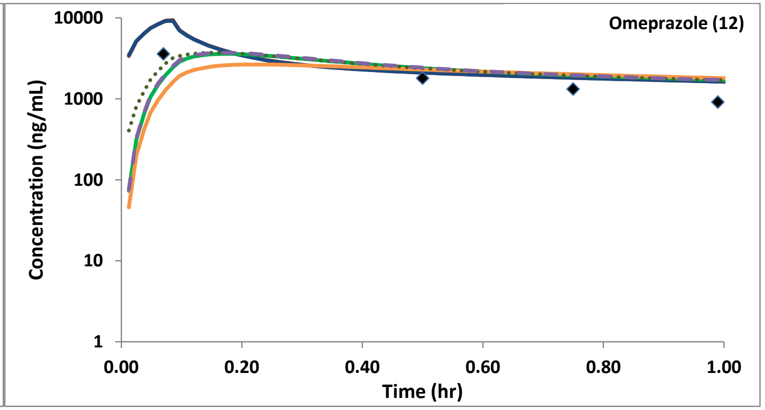


Rosiglitazone

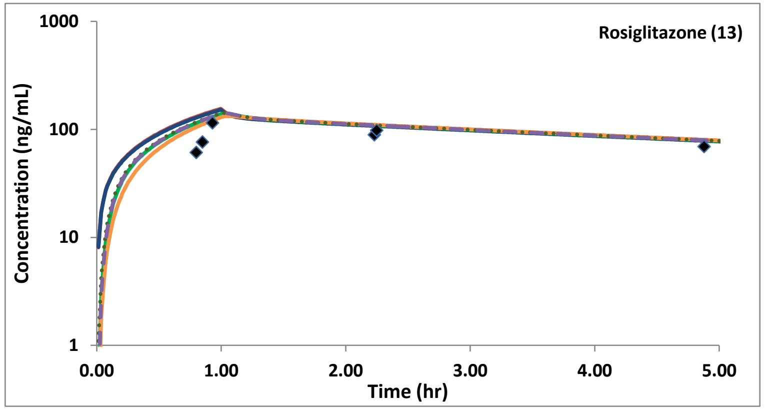

Theophylline

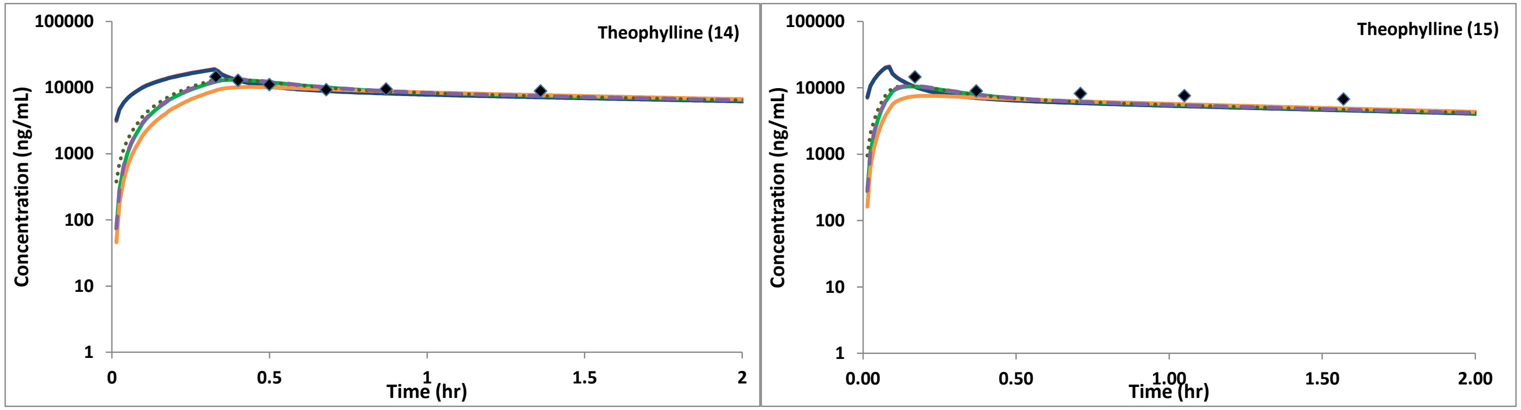

Supplement: Supplementary file 1 — (PDF 272 kb) [file 12248_2015_9796_MOESM1_ESM.pdf]
